# Pretreatment of garden biomass by alkali-assisted ultrasonication: effects on enzymatic hydrolysis and ultrastructural changes

**DOI:** 10.1186/2052-336X-12-76

**Published:** 2014-04-28

**Authors:** Jagdish Gabhane, SPM Prince William, Atul Narayan Vaidya, Duraisamy Anand, Satish Wate

**Affiliations:** 1 Solid and Hazardous Waste Management Division, National Environmental Engineering Research Institute, Nehru marg, Nagpur, Maharashtra, India

**Keywords:** Ultrasonication, Alkali pretreatment, Garden biomass, Enzymatic hydrolysis, Cellulosic ethanol, Ultra structural changes

## Abstract

The present investigation aims at studying the effectiveness of alkali-assisted ultrasonication on pretreatment of garden biomass (GB). Dry and powdered GB suspended in 1% NaOH was ultrasonicated for 15, 30 and 60 minutes at a frequency of 25 KHZ. The mode of action and effectiveness of alkali-assisted ultrasonication on GB was established through microscopic, scanning electron microscopic and X-ray diffraction studies. A perusal of results showed that alkali-assisted ultrasonication led to fibrillation of GB which ultimately facilitated enzymatic hydrolysis. The results also indicated that alkali-assisted ultrasonication is an efficient means of pretreatment of GB at moderate (45-50°C) working temperature and low (1%) concentration of alkali. The yield of reducing sugar after enzymatic hydrolysis increased almost six times as compared to control due to alkali-assisted ultrasonication.

## Introduction

Lignocellulosic substrates are potential sources for the production of ethanol through microbial intervention because they are abundant, cheap and renewable [1]. The process of conversion of lignocelluloses into glucose is through hydrolysis for which the lignin bound to xylan and glucomannan [2,3] is known to be a recalcitrant compound. Pretreatment is therefore necessary to delignify and facilitate the disruption of lignocellulosic moiety. Pretreatment alters the structure of cellulose and making it more accessible to the enzyme that convert carbohydrate polymer into fermentable sugar [4,5]. Thus, the general idea of pretreatment is to increase cellulose accessibility, which can be done by removing or altering hemicelluloses or lignin, decreasing the crystallinity of cellulose and increasing the surface area. Until now, the overall conversion of cellulose material into glucose has been hampered mostly by economic problems such as high cost of pretreatment. Hence, cost effective but efficient means of lignocellulosic pretreatment is crucial for viable production of cellulosic ethanol.

Generally, pretreatment methods are either physical or chemical. Some methods incorporate both effects [6]. Most of the conventional pretreatment processes utilize heat (thermal energy) to mediate bond breaking between molecules during chemical action. Different kinds of heating devices such as autoclave, microwave digesters, heating coils etc. are used in pretreatment processes. The energy consumption of these heating devices is normally high that ultimately affects cost effectiveness of cellulosic ethanol production. Dilute acid pretreatment has been widely studied because it is effective and relatively inexpensive [7]. Steam pretreatment (with pressure) is also one of the preferred methods of hydrolysis of lignocellulosic feed stocks. Steam pretreatment supplies moist heat under pressure that results in substantial breakdown of lignocellulosic structure, hydrolysis of hemicellulosic fraction, depolymerisation of lignin components and defibration [8]. Microwave irradiation has also been employed for lignocellulosic pretreatment [9,10]. Microwave generates thermal energy through dielectric heating [11] that alters the ultra structure of cellulose, degrade lignin and hemicellulose and increase the enzymatic susceptibility of reducing sugar [12].

Ultrasonication has seen wide application in cell crushing, removal of dead cell in surgery [13] and breaking filamentous algae in small pieces [14]. Sonication is the act of applying ultrasound energy to agitate the particles and to speed up dissolution of molecules by breaking the intermolecular interaction. Ultrasonication can be a promising alternative to conventional hydrolysis methods [15]. The ability of ultrasonication in degrading polymeric sequences has been well documented, particularly in synthetic materials dissolved in various solvents [16] and in extracting lignin and hemicellulose from lignocellulosic materials [17,18]. Some studies have suggested that pretreatment of lignocellulose substrates with ultrasonication could be useful for the intensification of bioconversion both in nature and under production condition [19].

Although reports [20-23] are available relating to the effects of ultrasonication on lignocellulosic disruption, there is paucity of information about the effectiveness of alkali-assisted ultrasonication, particularly on garden biomass. Garden biomass (GB), a potential cellulosic resource for bio energy production is commonly found in urban waste. It is rich in recalcitrant molecules such as cellulose and lignin, and relatively small amounts of saccharides, amino acids, and proteins. As GB is rich in cellulose, it can be used as a raw material for bio energy production after pretreatment.

Therefore, in the present study, we aimed to assess the efficacy of alkali-assisted ultrasonication on the pretreatment of GB. The major objectives of present investigation are: i) to study the effectiveness of alkali-assisted ultrasonication on ultra structure and delignification of GB ii) to assess the effectiveness of alkali-assisted ultrasonication on enzymatic hydrolysis and ethanol production.

## Material and methods

### Collection and processing of garden biomass

Garden biomass (GB) consisting mostly grasses (85-90%) of the species of *Cynodon ductylon* and *Elusine indicus* and small portion of weeds and fallen leaves was collected from the garden area of National Environmental Engineering Research Institute (NEERI) and sun dried for 2–3 days followed by oven drying at 70°C for about 92 hours. The dry GB was powdered using a pulverizer to pass a 1 mm sieve and kept in polyethylene sample containers inside a wooden cupboard at room temperature (28 ± 2°C) for further experiments. Known quantity of this powdered material was analyzed for initial composition and the remaining used for further experiments.

### Alkali –microwave pretreatment

Two gms of dry and powdered GB was taken in a 500 ml conical flask and suspended with 100 ml of 1% NaOH (W/V). The contents were mixed well and digested in a microwave digester (Ethos 900, Italy) at 180°C, 700 W for 30 minutes. The digested material was cooled and subjected for compositional analysis as per section 2.4.

### Alkali-assisted ultrasonication

Two gms of dry and powdered GB was taken in a 500 ml conical flask and was suspended with 100 ml of NaOH in three different (0.5, 1.0, and 5.0% ) concentrations (W/V). The contents were mixed well and sonicated for 15,30 and 60 minutes at 25 KHZ frequency with an effective ultrasonic power of 150 W using an ultrasonicator (Lark, USA). The control treatment received no sonication. The resultant liquid after ultrasonication was analyzed for reducing sugar, which was totaled, with the reducing sugar of enzymatic hydrolysis later on. The residue after repeated washings with de-ionized water was dried in an oven at 50°C for 48 hrs and analyzed for different parameters as per section 2.4.

### Compositional analysis of GB before and after pretreatment

Samples of GB (dry and powdered) collected before and after pretreatment were analyzed for cellulose content using its hydrolyzed residues by HNO_3_-ethanol method [24]. The hemicellulose content was analyzed by Liu method [24]. The lignin content of GB was estimated according to H_2_SO_4_ method [24]. The yield of reducing sugar after enzymatic hydrolysis was estimated by DNS method [25] using glucose as standard.

### Polarized light microscopic (PLM) and Scanning electron microscopic (SEM) studies on pretreated GB

The impact of alkali-assisted ultrasonication on surface morphology and ultra structure of both alkali-microwave and alkali-assisted ultrasonication pretreatment of GB was studied using polarized light microscope (Olympus, BX-80) and scanning electron microscope (JEOL-JED, Japan), respectively. The pretreated GB samples were dispersed in distilled water. The suspension was dropped and mounted on a glass slide and viewed through PLM. For scanning electron microscopic studies, samples of GB after pretreatment were mounted on glass slides, dried at 45°C in an oven before fixing it on stubs. The stubs were then coated with platinum by an ion sputter and imaged through SEM.

### XRD and crystallinity measurements

Samples of GB from both alkali-microwave and alkali-assisted ultrasonication pretreatments were examined by XRD using powder X- ray diffractometer [Model- Rigaku (Miniflex-ll)]. Approximately 50 mg of sample was taken and dried at 45-50°C in an oven for the removal of moisture. The dried sample was pressed in a specified sample holder and scanned at 2°/min from 2^ = 10° - 30°. The crystallinity index (Cr) was calculated from the XRD patterns by the empirical method proposed by Nelson and O’Conner, [26] using the following equation:

$$\mathrm{Cr}\;I\;=\;\frac{\mathrm{Ic}r-I\mathrm{am}}{\mathrm{Ic}r}$$

Where Cr I is the crystallity index, Ic*r* is maximum diffraction intensity at peak position 2^ ~ 22.6° and I*am* is the intensity at 2^ = 18.7°

### Enzymatic hydrolysis

The residue of GB after pretreatment was washed thoroughly with de-ionized water and enzymatically hydrolyzed using a commercial cellulase (pure) enzyme, ONOZUKAR-10 procured from Hi-Media, India. The solid loading rate for enzymatic hydrolysis was 6 g/100 ml (0.05 M citrate buffer) with an enzyme loading rate of 50 mg (50 FPU)/g of GB. The pH during enzymatic hydrolysis was 4.8 and the temperature maintained at 50°C using a shaker incubator operated at 150 rpm for 48 hrs. 2.5 mg of Tetracycline was also added to avoid microbial contamination during enzymatic hydrolysis.

### Statistical analysis

All experiments were replicated thrice and mean values with standard deviation presented in tables. The characterization of GB before and after pretreatments were statistically analyzed using one way ANOVA and Duncan’s Multiple Range Test (DMRT) using SPSS software (version 11.5)

## Results and discussion

### Characterization of garden biomass (GB)

GB used in the present investigation contained mostly grasses and small fraction of weeds and fallen leaves. The characterization of GB revealed that it contains 44.03% of cellulose, 28.55% of hemicellulose and 24.46% of lignin Table 1. The concentration of lignocellulosic moiety in GB as compared to other lignocellulosic materials such as vegetable waste [27] and municipal solid waste [28] is high. Generally, raw materials with good amount of cellulose and hemicelluloses are preferred for cellulosic ethanol production.

**Table 1:** Composition of garden biomass expressed as % of dry matter

**Sr. no**	**Parameters**	**Values (%)**
1	Total organic matter	85.70
2	Organic carbon	45.13
3	Total nitrogen	1.16
4	Ash content	12.58
5	C/N ratio	38.90
6	Cellulose	44.03
7	Hemicellulose	28.55
8	Lignin	24.46
9	Sodium	1.98
10	Potassium	1.17
11	Phosphorous	0.98

### Effectiveness of alkali concentration and reaction (ultrasonication) time on enzymatic hydrolysis of GB

Three alkali (NaOH) concentrations (0.5, 1.0, and 5.0% (w/v)) were tested against three (15, 30, and 60 minutes) different reaction (ultrasonication) times to find out the best effective combination (BEC) of alkali concentration and reaction (ultrasonication) time for enzymatic hydrolysis. BEC was fixed based on reducing sugar yield (Table 2) after enzymatic hydrolysis. Accordingly, an alkali concentration of 1% with 60 minutes of ultrasonication was found to be the BEC as it yielded maximum reducing sugar from GB. Although 60 minutes of ultrasonication is too long for an economic pretreatment process, it should be noted that alkali assisted ultrasonication even at 15 minutes of reaction time was effective and increased reducing sugar yield compared to control, however, the maximum yield was observed at 60 minutes.

**Table 2:** Effectiveness of alkali concentration and sonication time on reducing sugar yield after enzymatic hydrolysis

**Time (min) and concentration (%) of alkali**	**Reducing sugar yield after enzymatic hydrolysis**	**% increase/decrease in reducing sugar concentration**
	**(%, w/w)**	
00, 0.0	5.40 ± 0.65 a	---
00, 0.5	26.53 ± 0.76 c	391.29
00, 1.0	32.76 ± 1.92 d	506.66
00, 5.0	39.26 ± 0.78 ef	627.03
15, 0.0	7.64 ± 2.33 ab	41.48
15, 0.5	31.05 ± 3.34 d	475.00
15, 1.0	37.71 ± 1.23 e	598.33
15, 5.0	41.63 ± 0.62 f	670.92
30, 0.0	8.41 ± 1.49 ab	55.74
30, 0.5	36.54 ± 1.94 e	576.66
30, 1.0	48.13 ± 1.57 g	791.29
30, 5.0	52.32 ± 0.80 h	868.88
60, 0.0	9.55 ± 1.58 b	76.85
60, 0.5	42.42 ± 1.68 f	685.55
60, 1.0	59.56 ± 2.60 j	1002.96
60, 5.0	56.40 ± 3.43 i	944.44

Data are means ± SD (n=3).

Means followed by the same letter in a column are not significantly different at P ≤0.05 by DMRT.

The increase in reducing sugar yield was significantly high only when ultrasonication was assisted by alkali. On the other hand, pretreatment of GB with either with alkali or ultrasonication alone did not cause any increase in reducing sugar yield. Ultrasonication of GB for 60 minutes without alkali yielded 9.55% of reducing sugar after enzymatic hydrolysis, whereas ultrasonication with alkali (1%) yielded 59.56% of reducing sugar, more than six fold increase in reducing sugar over ultrasonication alone treatment. Similarly, 1% alkali alone yielded 32.76% of reducing sugar which is much lesser than the yield (59.56) of alkali assisted ultrasonication.

Alkali (NaOH) has been extensively studied and proven effective for pretreatment of wheat and rice straw [29,30]. The main effect of sodium hydroxide pretreatment on lignocellulosic biomass is delignification by breaking the ester bonds cross-linking lignin and xylan, thus increasing the porosity of biomass [31]. However, the relative rate of degradation (peeling) and stabilization (stopping) depends on conditions such as the nature and the concentration of the alkali and on temperature. Generally, stabilization is favoured at high temperature and higher alkali concentration [32]. Varga et al. [33] reported 95% reduction in lignin content as a result of pretreatment of corn stover with 10% NaOH for 1 h in the autoclave. Zhao et al. [34] found 2% of NaOH as the effective dosage for alkaline pretreatment of spruce at low temperature. Similarly, Silverstein et al. [35] also found a maximum of 65.63% reduction in lignin content with 2% NaOH treatment for 90 min at 121°C/15 psi. He further stated that an increase in the concentration of NaOH significantly improved delignification at all combinations of temperature and time. In the present investigation, we found 45% (Table 3) reduction in lignin concentration at 1% NaOH with 60 minutes of ultrasonication. This when compared to the previous reports is slightly low, may be because of the low temperature (45-50°C) involved in alkali-assisted ultrasonication pretreatment. Nevertheless, pretreatment at low temperature would be beneficial particularly for the reason that elevated temperature can cause greater degradation of hemicellulosic sugars leading to the conversion of reducing sugar into other compounds such as HMF etc.

**Table 3:** Initial and final characterization of garden biomass after alkali- microwave and alkali-sonication pretreatments

**Parameter**	**Initial concentration (%)**	**Final concentration (%)**		
		**Alkali alone**	**Alkali + Microwave**	**Alkali + Sonication**
Cellulose	44.03 ± 1.39 a	66.25 ± 2.83 b	73.08 ± 0.77 c	77.28 ± 0.85 d
Hemi-Cellulose	28.55 ± 3.19 c	12.85 ± 0.69 b	7.19 ± 0.32 a	8.02 ± 0.85 a
Lignin	24.46 ± 0.64 d	19.80 ± 0.82 c	15.86 ± 0.87 b	13.39 ± 0.69 a

Data are means ± SD (n=3).

Means followed by the same letter in a column are not significantly different at P ≤0.05 by DMRT.

### Effects of alkali-assisted ultrasonication on ultra structural changes of garden biomass

**Figure 1:**
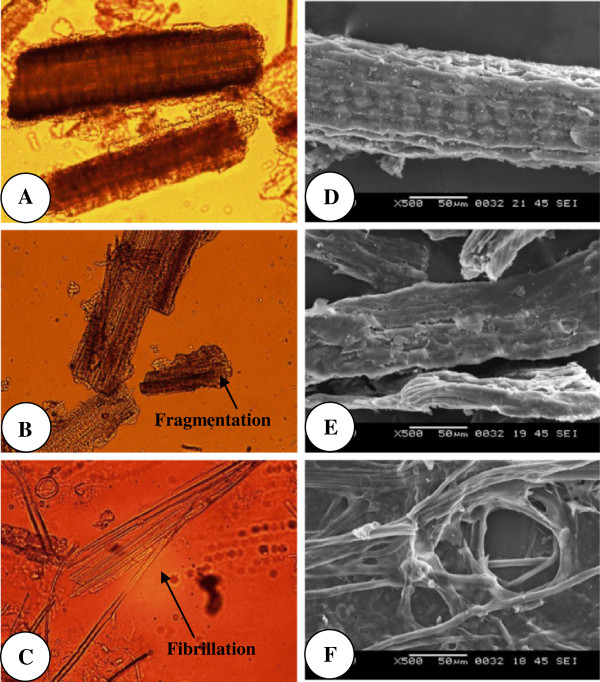
**Light and scanning electron microscopic views of control and pretreated GB. A**: Polarized light microscopic view of raw garden biomass. **B**: Polarized light microscopic view of alkali-microwave pretreated garden biomass showing fragmentation. **C**: Polarized light microscopic view of alkali-sonication pretreated garden biomass showing fibrillation. **D**: Scanning electron microscopic view (500 X) of untreated garden biomass. **E**: Scanning electron microscopic view (500 X) of garden biomass pretreated with alkali (1% NaOH)-sonication. **F**: Scanning electron microscopic view (500 X) of garden biomass pretreated with alkali (1% NaOH)-microwave.

The mode of action and impact of alkali-assisted ultrasonication on garden biomass was analyzed through microscopic studies using both polarized light microscope (PLM) and scanning electron microscope (SEM). For a clear understanding and precise evaluation of the impact, we compared the ultra structural changes in GB due to alkali-assisted ultrasonication with alkali-microwave treatment. Figure 1A-1F illustrate the results of microscopic studies of both alkali-microwave and alkali-assisted ultrasonication pretreatments. While comparing the mode of action of both alkali-microwave and alkali- assisted ultrasonication pretreatments, it was found that alkali-microwave at higher temperatures (200°C) developed fragmentation (Figure 1B & 1E) in GB, whereas, alkali- assisted ultrasonication resulted in defibration and fibrillation (Figure 1C and 1F). Defibration of GB is mainly because of alkali action that removed lignin from lignocellulose and sonication forked cellulose fibers into fine fibrils (Fibrillation). Such kind of splitting of cellulose fibers due to sonication has already been documented. Cheng et al. [36] reported that after high intensity ultrasonic treatment, most particles of Avicel cellulose were split into smaller fibrils. Tang and Liang [37] also suggested that ultrasonication can crack the cell wall, dislocating the secondary wall of the middle layer and resulting in fibrillation. Further, it was observed that ultrasonication even at longer duration did not produce any chaotic damage on tissue structure; instead, it resulted in fibrillation (Figure 1C & 1F) facilitating the enzymatic hydrolysis. Whereas, alkali-microwave treatment at high temperature led to complete tissue collapse (Figure 1B & 1E), which may hinder enzymatic hydrolysis process as it leaves less space of enzyme action on substrate.

### Effect of alkali-assisted ultrasonication on crystallinity of cellulose in garden biomass

The measurement of crystallinity index is important because ultrasonic treatment of cellulose generally cause a strong decrease in the degree of polymerization [38]. A perusal of results showed an increase (0.828) in CrI of alkali-ultrasonicated GB over the control (0.729). However, comparing to alkali-microwave (0.848) pretreatment, this is slightly low, might be because of high temperature (180°C) involved in alkali-microwave pretreatment that effectively converted maximum of amorphous fraction of cellulose into reducing sugars and exposing the crystalline fraction more prominently.

## Conclusion

Based on results, it may be concluded that alkali-assisted ultrasonication is one of the effective methods of pretreatment for GB as it increases the net yield of reducing sugar after enzymatic hydrolysis. The microscopic studies further revealed that alkali-assisted ultrasonication caused fibrillation of cellulosic moiety, which ultimately favored enzymatic hydrolysis process. Alkali-ultrasonication was carried out at moderate temperature, at which further degradation of reducing sugar is not possible, an advantageous condition for alcohol fermentation.

## Abbreviations

GB: Garden biomass; FPU: Filter paper unit; PLM: Polarized light microscope; SEM: Scanning electron microscope; CrI: Crystallinity index.

## Competing interests

The authors declare that they have no competing interests.

## Authors’ contributions

JG and SPMPW are the main investigators, performed all experimental work, data analysis and paper writing. DA carried out statistical analysis, ANV and SRW provided all essential supports. All authors read and approved the final manuscript.

## Authors’ information

Solid and Hazardous Waste Management Division, National Environmental Engineering Research Institute, Nehru marg, Nagpur, Maharashtra, INDIA.
